# Electronic, Magnetic, and Optical Performances of Non-Metals Doped Silicon Carbide

**DOI:** 10.3389/fchem.2022.898174

**Published:** 2022-04-19

**Authors:** Lin Zhang, Zhen Cui

**Affiliations:** ^1^ School of Science, Xi’an University of Technology, Xi’an, China; ^2^ School of Automation and Information Engineering, Xi’an University of Technology, Xi’an, China

**Keywords:** silicon carbide, magnetism, non-metals, electronic and optical properties, work function (WF)

## Abstract

The configurations of nine different non-metals doped silicon carbide (NM-SiC) were structured by using the density functional theory (DFT). The magnetic, electronic, and optical properties of each NM-SiC are investigated at the most stable structure with the maximum binding energy. Although the O-, Si-, and S-SiC systems are still non-magnetic semiconductors, the N- and P-SiC systems have the properties of the magnetic semiconductors. The H-, F-, and Cl-SiC systems exhibit the half-metal behaviors, while the B-SiC system converts to magnetic metal. The redistribution of charges occurs between non-metals atoms and adjacent C atoms. For the same doping position, the more charges are transferred, the greater the binding energy of the NM-SiC system. The work function of the NM-SiC systems is also adjusted by the doping of NM atoms, and achieves the minimum 3.70 eV in the P-SiC, just 77.1% of the original SiC. The absorption spectrum of the NM-SiC systems occurs red-shift in the ultraviolet light region, accompanying the decrease of absorption coefficient. These adjustable magnetic, electronic, and optical performances of NM-SiC expand the application fields of two-dimensional (2D) SiC, especially in designing field emission and spintronics devices.

## Introduction

The last few decades, 2D materials (Li and Kaner, 2008; Liu et al., 2014; Zhong et al., 2019; Luo et al., 2021) have been extensively used in optoelectronics (Stankovich et al., 2006; Bratschitsch, 2014; Cui et al., 2021a; Sun et al., 2021), catalysis (Ziletti et al., 2015; Wang et al., 2018), spintronic devices (Komsa et al., 2012; Sun et al., 2017a; Li et al., 2021), energy conversion (Pospischil et al., 2014; Cui et al., 2020a; Sun et al., 2019; Sun and Schwingenschlögl, 2020), and gas sensing (Kooti et al., 2019; Cui et al., 2020b) for their unique structural, optical, electronic and magnetic properties (Eddy and Gaskill, 2009; Castelletto et al., 2014; Yuan et al., 2018; Sun and Schwingenschlögl, 2021). Considering the high thermal capability and carrier mobility of bulk SiC (Mélinon et al., 2007; Susi et al., 2017; Ferdous et al., 2019), the theoretical and experimental research on 2D SiC has aroused significant attention (Hsueh et al., 2011; Chowdhury et al., 2017; Chabi and Kadel, 2020). Although density functional theory predicts that 2D SiC has a graphene-like structure with alternating Si and C atoms (Lambrecht et al., 1993; Bekaroglu et al., 2010), the difficulty in stable 2D SiC synthesis has put the research on a standstill (Lin, 2012). Recently, Chabi et al. (2021) fabricated the stable 2D SiC monolayer by wet exfoliation, and predicted the potential applications in integrated microelectronics circuits and light-emitting devices. This work promotes the enthusiasm for the 2D-SiC and SiC-based systems.

Earlier studies on other 2D materials provide much valuable guidance for the actual application of 2D SiC. Both doping and adsorption are shown to be the effective methods to regulate the 2D material properties (Tang et al., 2018; Cui et al., 2021b). For instance, in transition metal (TM) doping, the orbital hybridization between the TM atom and the substituted atom arouses a robust local magnetization in a 2D material, stimulates the design of spintronics devices (Sun et al., 2017b; Yuan et al., 2020). This potent magnetism has been predicted when the Si or the C atom in SiC system is substitutes by TM atoms, such as, Mn atom (Bezi Javan, 2016; Luo et al., 2017; Wu et al., 2019).

When 2D materials are absorbed or doped by non-metals (NM) atoms, such as, H, N, O, and Cl et al., the orbital hybridization between the NM atom and the substituted atom will not only cause a magnetization in the host 2D material, but also low down the work function which effects the electrons-emitting ability (He et al., 2010; Luo and Shen, 2018). Moreover, the absorption spectrum of 2D material can be tuned to improve the photocatalysis ability by the injection of NM atoms (Cui et al., 2021c). All these predict that NM-SiC systems can be used in spintronics, field emitters, and photocatalysis regions. To the best of our knowledge, the magnetic, electrical, and optical properties of NM-SiC are still unclear. To maximize the 2D-SiC advantage, we investigated the magnetic, electronic, and optical behaviors of nine stable NM doped SiC systematically. Our results show that the properties of these NM-SiC system are changed after the doping of NM atoms. Although the O-, Si-, S-SiC systems are still non-magnetic semiconductors, the N- and P-SiC systems exhibit the properties of the magnetic semiconductors. The H-, F-, and Cl-SiC systems emerge the half-metal behaviors, while the B-SiC is converted to magnetic metal. The work function of the P-SiC is adjusted as low as 3.7 eV, just 77.1% of the 2D-SiC. The red-shift of the absorption spectrum occurs in the ultraviolet light region. These results demonstrate the potential application of NM-SiC in spintronics and field electron-emitting devices.

## Computations Details

**FIGURE 1:**
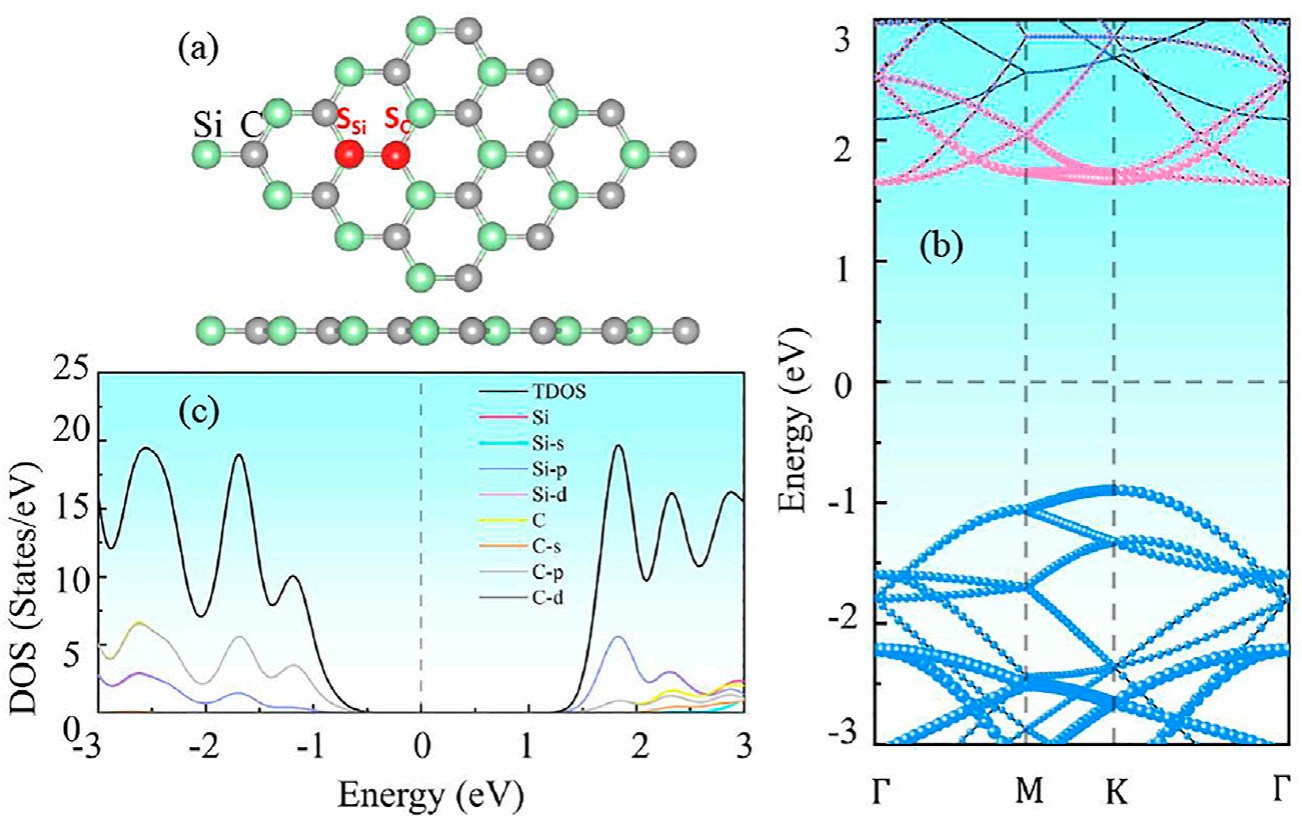
The **(A)** crystal structure, **(B)** energy band structure, and **(C)** density of states of intrinsic 2D SiC.

Vienna *Ab Initio* Simulation Package was employed to investigate the characteristics of non-metal doped SiC, such as, the band structures, bonding energy, charge transfer, magnetic properties, and work function (Kresse and Furthmüller, 1996). The exchange-related interactions were expressed as the Perdew–Burke–Ernzerhof functions (PBE) based on Generalized gradient approximation (GGA) (Kresse and Joubert, 1999; Perdew et al., 1996). The DFT-D3 of Grimme was used to resolve the weak dispersion forces (Heyd et al., 2003). The cut-off energy of the plane wave was chosen at 550 eV. A 4 × 4×1 NM-SiC supercell substituted by one non-metal atom is structured as depicted in Figure 1A. The Brillouin zone consists of 3 × 3×1 Monkhorst-Pack k-point grids (Grimme et al., 2010). A 15 Å vacuum layer was set in the vertical direction of SiC. All systems are complete relaxed to ensure that the systems reach the most stable states, where the total energy change is lower than 10^−5^ eV/atom and the Hellmann-Feynman force on each atom is less than 0.01 eV/Å. Then the frequency-dependent dielectric response theory is used to investigate the optical properties of the NM-SiC systems in random-phase approximation (RPA) (Hybertsen and Louie, 1986).

## Results and Discussion

The pristine SiC exhibits a complete planar structure, the calculated lattice parameter is 3.10 Å, as shown in Figure 1A. Figure 1B illustrates that 2D SiC is a direct band semiconductor with a gap of 2.52 eV. The density of states (DOS) diagram in Figure 1C demonstrates that the conduction band of SiC is determined by the p orbit of Si, while the valence band is contributed by the p orbit of C primarily. All these results agree with the previous report (Chabi et al., 2021), which confirms the validity of our computational models.

Binding energy is an important parameter reflecting the structural stability of system, and described as follows,

$$E_{\text{b}}=E_{\text{NM}+\text{SiC}}-\left(E_{\text{SiC}}+E_{\text{NM}}\right)$$ (1)

Where *E*_b_ is the bonding energy, corresponding to the energy difference of the systems before and after doping. *E*_NM+SiC_ denotes the energy of the non-metal doped SiC, *E*_SiC_ represents the energy of the original SiC with one vacancy, *E*_NM_ is the energy of the doping atom, respectively. The negative binding energy implies that the NM-SiC system has better stability than that before. A larger value indicates a more stable system. The binding energies were calculated at all possible high symmetry doping positions. For 2D SiC, the two possible substituted sites are the S_Si_ (substitute Si atom), and the S_C_ (substitute C atom).

Table 1 lists the parameters of the nine NM-SiC systems with most robust *E*_b_. All the NM-SiC systems present high stability. The steadiest doping position of the NM-SiC systems varies with the doping atoms. The atoms, such as, H, B, N, Si, P, and S, prefer to locate at the position S_Si_, while the O, F, and Cl atoms select the position S_C_. For those configurations structured at the position S_Si_, the B-SiC system exhibits the most potent binding energy. For those configurations at the position S_C_, the O-SiC system has the largest binding energy. For the same doping position, the stronger the binding energy, the greater the interaction of the NM with the neighboring atoms. The following researches on NM-SiC systems are explored on these steadiest configurations.

**TABLE 1:** The doping position, binding energy (*E*_b_), charge transfer (*C*), band gap (*E*_g_), and magnetic moment (*M*_total_) of the NM-SiC systems.

Doping style	Position	*E*_b_	*C* (e)	*M*_total_ (μB)	*E*_g_ (eV)
H	S_Si_	−4.874	−0.188	2.982	0
B	S_Si_	−15.006	−1.843	0.553	0
N	S_Si_	−14.627	+0.832	1	1.250
O	S_C_	−10.136	+1.692	0	1.959
F	S_C_	−5.735	+0.812	1	0
Si	S_Si_	−14.682	−2.516	0	2.541
P	S_Si_	−13.498	−1.772	1	2.483
S	S_Si_	−10.995	−0.450	0	2.130
Cl	S_C_	−4.209	+0.596	1	0

**FIGURE 2:**
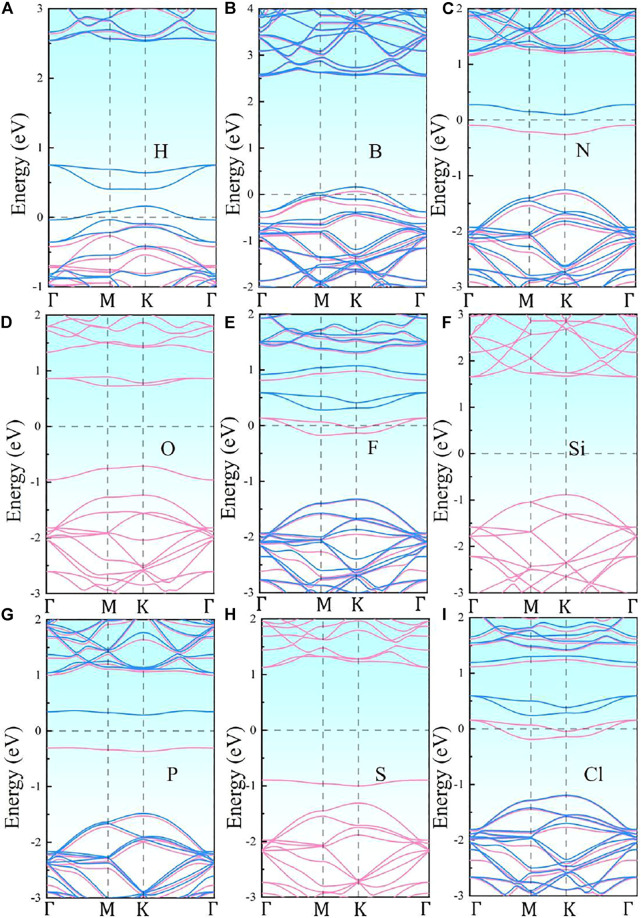
The band structures of NM-SiC systems: **(A)** H-SiC, **(B)** B-SiC, **(C)** N-SiC, **(D)** O-SiC, **(E)** F-SiC, **(F)** Si-SiC, **(G)** P-SiC, **(H)** S-SiC, **(I)** Cl-SiC. The pink lines and the blue lines represent the spin-up, and the spin-down components of energy levels, respectively. The Fermi level is shifted to zero.

The band structures of the nine different NM-SiC systems are illustrated in Figure 2. It can be seen that the energy band structures of NM-SiC systems are similar to the original SiC system to a large extent. The appearance of the impurity levels causes the change in the SiC band structure. The O-, Si-, and S-SiC are still nonmagnetic semiconductors, and the corresponding band gaps are 1.959 eV (O), 2.541 eV (Si), and 2.130 eV (S), respectively. The H-, B-, N-, F-, P-, and Cl-SiC systems exhibit magnetism for the asymmetry between the spin-up and spin-down components of the energy levels. Among them, the B-SiC is converted to magnetic metal because that Fermi level intersects with both the spin-up and the spin-down components. The H-, F-, and Cl-SiC systems exhibit the half-metal behaviors, and the Fermi level only intersects with the spin-down component. The N- and P-SiC systems convert to magnetic semiconductors with band gaps of 1.250 eV (N) and 2.483 eV (P), respectively.

To analyze the magnetism of NM-SiC systems, the spin-polarized charge density *ρ* is calculated,

$$\rho =\rho_{\text{up}}-\rho_{\text{down}}$$ (2)

where *ρ*_up_ and *ρ*_down_ are the up spin-polarized charge density and the down spin-polarized charge density, respectively.

**FIGURE 3:**
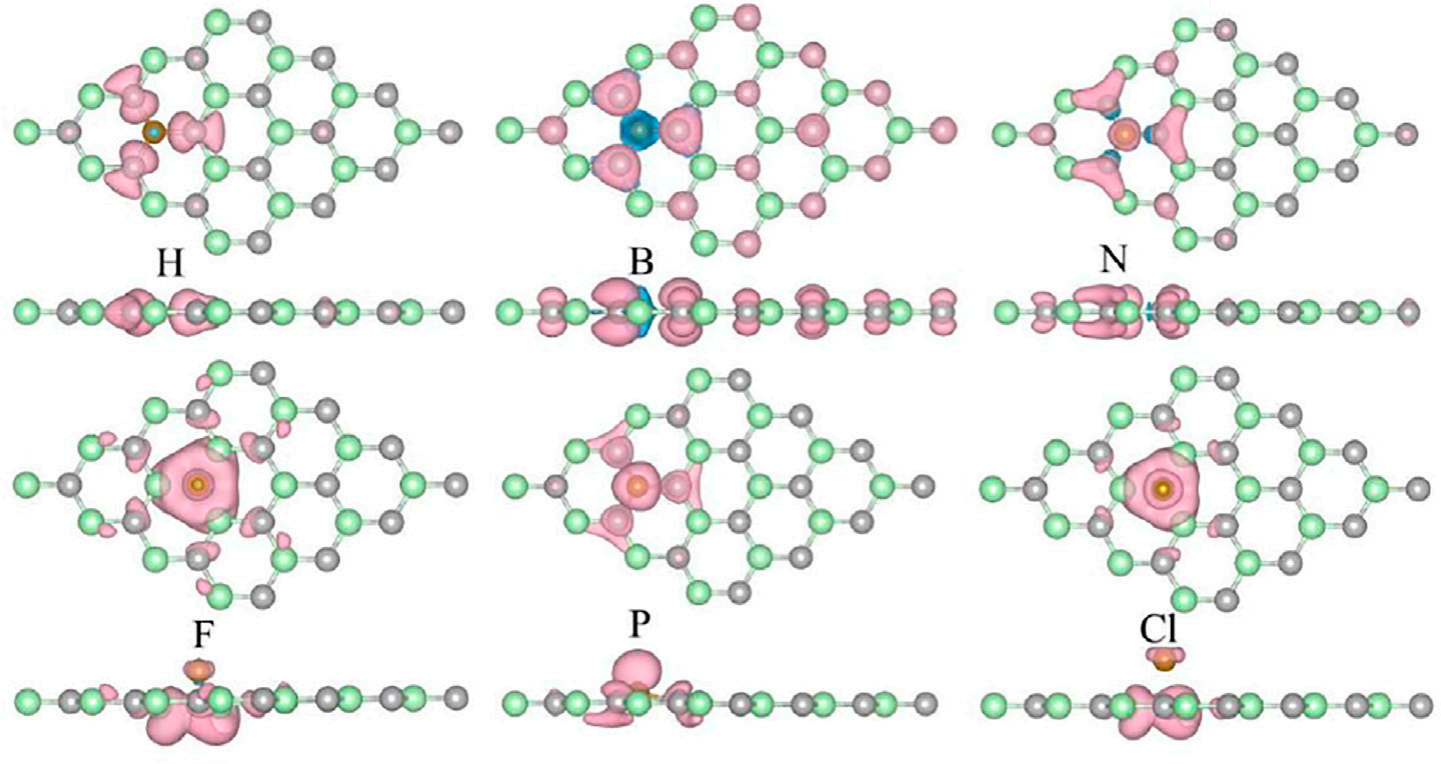
The spin-polarized charge density of the metal-doped SiC system. The pink and blue areas represent the contribution of the spin-up, and the spin-down components, respectively. The isovalue is set to 0.001 e/Å^3^.

Figure 3 illustrates the spin-polarized charge density distributions of the magnetism H-, B-, N-, F-, P-, and Cl-SiC systems. It can be seen that the spin-polarized charge occurs near the doping atoms and the neighboring atoms. The corresponding magnetism is primarily caused by the doping NM atoms, while the adjacent atoms make a smaller contribution. For metals, the magnetic moment is calculated as 2.982 μB (H), 0.553 μB (B), 1 μB (F), and 1 μB (Cl). When we compare the semiconductor N-,O-, Si, P, and S-SiC systems, it can be seen that the magnetic moment exhibits a regular change with atomic number, 1 μB (N), 0 μB (O), 0 μB (Si), 1 μB (P), and 0 μB (S), respectively. The doping atoms with the same family cause the same magnetic moment, such as, N and P, O and S. This phenomenon can be explained by comparing with the stable non-magnetic SiC. The valence electron difference between the doping atom and the substituted atom are 1 (N), 2 (O), 0 (Si), 1 (P), and 2 (S), respectively. When equal amounts of charge refill the vacancy of the substituted atom, the redundant electrons begin to fill the impurity level with the exclusion principle. And then the corresponding magnetism is formed. This adjustable magnetism expands the application of NM-SiC in nano-spintronics devices.

**FIGURE 4:**
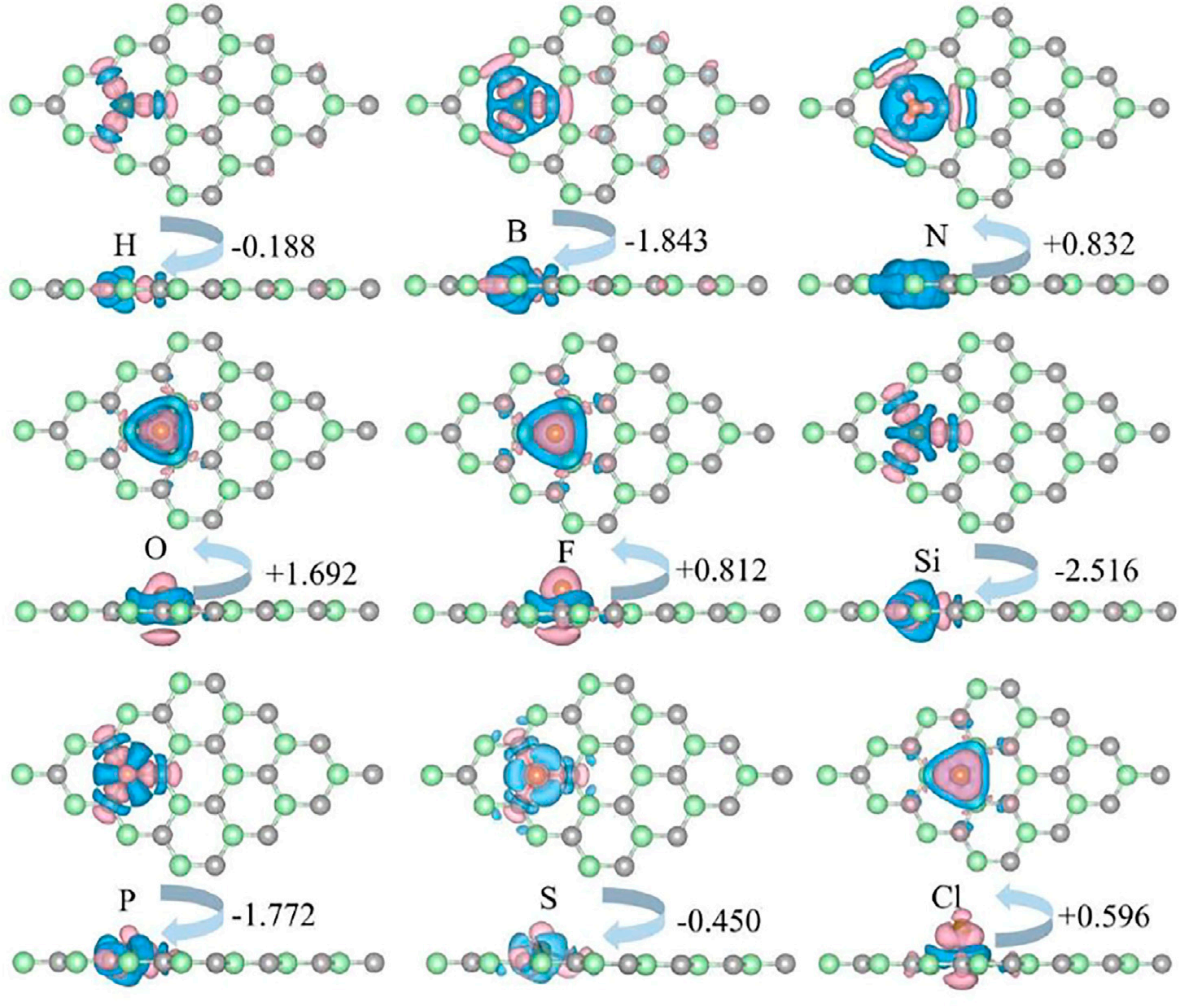
The charge density difference of the NM-SiC system. The isovalue is set to 0.001 e/Å^3^. The pink and the blue regions represent the gain, and the loss of the charge. The negative sign indicates the NM atoms act as charge donors, while the positive sign indicates the NM atoms act as charge acceptors.

The injection of impurity atoms causes the redistribution of charges between the doping and the substituted atoms, which leads to the change in electronic properties of 2D SiC. The charge density difference (CDD) is calculated as follows,

Δρ=ρTotal−(ρ+SiCρNM) (3)

where *ρ*_Total_, *ρ*_SiC_ and *ρ*_NM_ represent the charge density of the NM-SiC, the original SiC, and the NM atom, respectively. ∆*ρ* is the charge density difference of the systems before and after doping. As depicted in Figure 4, the charge transfer occurs between the doping NM atom and the neighboring atoms. The charge transfer is calculated by Bader charges (Henkelman et al., 2006; Sanville et al., 2007), which are, -0.188|e| (H), -1.843|e| (B), +0.832|e| (N), +1.692|e| (O), +0.812|e| (F), −2.516|e| (Si), −1.772|e| (P), -0.450|e| (S), and +0.596|e| (Cl), respectively. The negative sign implies the loss of charge, while the positive sign appears as the obtain of charge. For the N-, O-, F-, and Cl-SiC systems, NM atoms act as acceptors obtaining some charge. For the H-, B-, Si-, P-, and S-SiC systems, NM atoms act as charge donors. A larger Bader charge indicates a more potent charge transfer. For the same doping position, a larger charge transfer indicates a stronger covalent bond interaction between the NM atom and the neighboring atoms (Pino-Rios et al., 2020), corresponding to a more potent binding energy of NM-SiC, as listed in Table 1.

**FIGURE 5:**
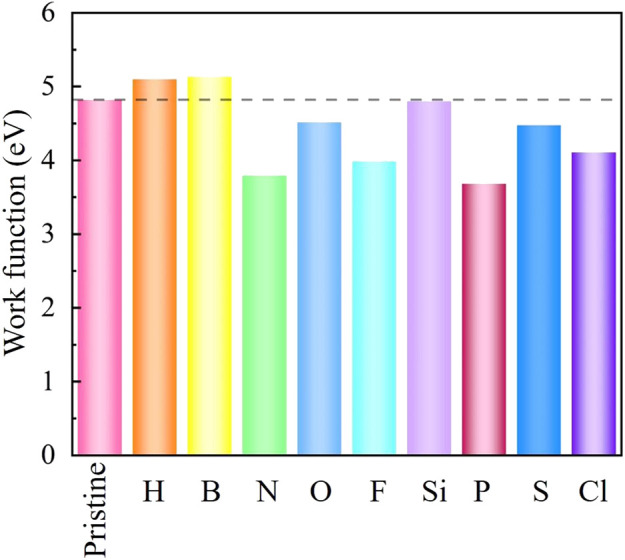
The work function of the intrinsic SiC and NM-SiC systems.

As an important electrical parameter of 2D SiC, work function is described as the minimum energy required to make the internal electrons escape into the vacuum. A smaller work function implies a stronger emitting electron capacity. As shown in Figure 5, although the work function of the pristine SiC is 4.82 eV, the work function of NM-SiC systems is regulated between 3.70 and 5.15 eV, covering the range of the traditional field electron emission devices 4.50–5.15 eV (Yu et al., 2009; Jiao et al., 2012; Cai et al., 2014; Soo et al., 2014). The work function of the H- and B-SiC are larger than the pristine SiC, while the N-, O-, F-, P-, S-, and Cl-SiC systems are lower than the pristine SiC. The work function reaches the minimum 3.70 eV in the P-SiC, just 77.1% of the pristine SiC. The NM-SiC systems exhibit the potential in the design of field electron emitter.

**FIGURE 6:**
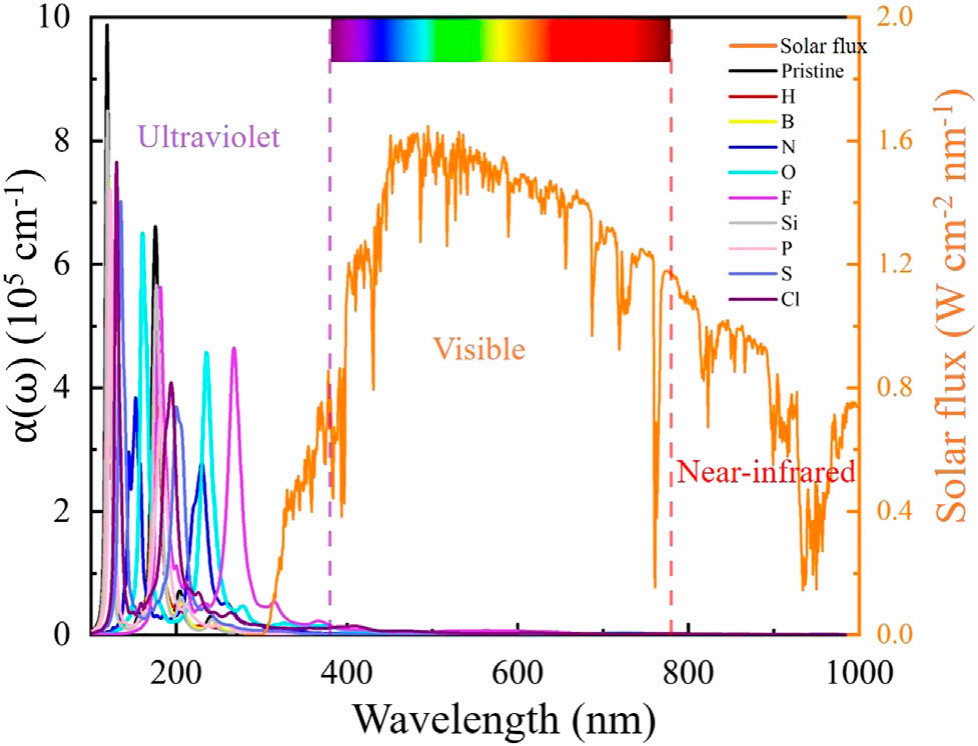
The absorption spectrum of intrinsic SiC and NM-SiC systems.

Moreover, the changes in the optical absorption spectrum were investigated. As shown in Figure 6, the intrinsic SiC has two acute absorption peaks in the ultraviolet region, and the higher is located at 50.1 nm wavelength with an absorption coefficient of 9.8×10^5^ cm^−1^. The optical absorption spectrum of NM-SiC varies with the doping atoms. The absorption spectrum occurs red-shift in the ultraviolet light region, and obtains the largest deviation in the P-SiC system.^1^ The absorption coefficient of NM-SiC experiences a greatly decrease. The absorption properties of NM-SiC systems are adjusted by the doping of the NM atoms.

## Conclusion

The magnetic, electronic, and optical properties of nine NM-SiC systems were investigated by density functional theory systematically. The steadiest configuration of each NM-SiC system is confirmed at the doping position with the maximum binding energy. Our results show that the optimal doping position varies with the NM atoms. The atoms H, B, N, Si, P, and S prefer to locate at the position S_Si_, while the O, F, and Cl select the position S_C_. The doping of the NM atoms causes the change in the properties of SiC system. Although the O-, S-, and Si-SiC systems are still non-magnetic semiconductors, the N- and P-SiC systems have the properties of the magnetic semiconductors. The H-, F-, and Cl-SiC systems exhibit the half-metal behaviors, while the B-SiC system converts to magnetic metal. The charges transfer between the NM atoms and the adjacent C atoms. For the same doping position, a more significant charge transfer indicates stronger binding energy of NM-SiC. The redistribution of charge causes the change in the work function of NM-SiC. In the N-, O-, F-, P-, S-, and Cl-SiC systems, the work function exhibits a decrease, and it achieves the minimum 3.70 eV in the P-SiC, just 77.1% of the pristine SiC. Compared with the pristine SiC, the absorption peaks intensity of each NM-SiC is decreased. The absorption spectrum occurs red-shift in the ultraviolet light region. All these results indicate the possibility of tuning the electronic, magnetic performance of NM-SiC by the doping of suitable non-metal atoms. This study provides theoretical guidance for designing 2D SiC-based spintronics and field emission devices.

## Data Availability

The original contributions presented in the study are included in the article/Supplementary Material, further inquiries can be directed to the corresponding author.

## Footnotes

1 The AM 1.5G spectrum was taken from the NREL website, http://rredc.nrel.gov/solar/spectra/am.1.5.
